# Absolute dynamic and relative static: the relationship of glycolysis and OXPHOS in cancer development

**DOI:** 10.1038/s41420-026-02992-5

**Published:** 2026-03-05

**Authors:** Xingting Bao, Boru Hou, Zhong Guo, Lei Song, Hailin Chen, Qian Zheng, Yongqing Zhao, Dandan Gao, Chenlong Fan, Xiaoyang Xiong, Chao Sun, Jin Zhao

**Affiliations:** 1 Medical College of Northwest Minzu University, Lanzhou, China; 2 Department of Neurosurgery, Lanzhou University Second Hospital, Lanzhou, China; 3 School of Life Sciences and Engineering of Northwest Minzu University, Lanzhou, China; 4 Department of Medical Physics, Institute of Modern Physics, Chinese Academy of Sciences, Lanzhou, China

**Keywords:** Cancer metabolism, Cancer microenvironment

## Abstract

For a significant period following the postulation of the Warburg effect, mitochondrial dysfunction and aerobic glycolysis were commonly accepted as the defining features of cancer. Currently, a deeper understanding of tumor metabolism has demonstrated that the energy phenotype of tumor cells is not solely glycolytic. Most cancer cells possess active mitochondria and still maintain the ability to undergo oxidative phosphorylation (OXPHOS) and utilize the tricarboxylic acid (TCA) cycle to support tumor growth. In this review, we examine the choice of energy supply pathways in tumor cells in both static and dynamic contexts. From a static standpoint, tumors contain cells that rely on glycolysis or OXPHOS for energy supply and demonstrate metabolic heterogeneity. Additionally, the simultaneous operation of glycolysis and OXPHOS establishes metabolic symbiosis. In contrast, cancer cells can also exhibit metabolic plasticity by dynamically shifting between glycolysis and OXPHOS to support tumor growth. This process is influenced by a variety of factors, such as the ever-changing tumor microenvironment, specific biological activities of tumor cells, and the effects of drug therapies. The relationship between glycolysis and OXPHOS suggests that in the process of cancer development, the stable state of energy metabolism is temporary, while the dynamic changes in energy metabolism are eternal, which is in line with the category of dialectical materialism and provides us with a new perspective for treating cancer.

## Facts

- Tumor heterogeneity results in the presence of various energy metabolism pathways, and the reprogramming of high-level energy metabolism enables tumors to interchange between these pathways.
- The flexible and diverse energy metabolism of tumors ensures an abundant energy supply while also providing the necessary metabolic products for the rapid growth of tumors.
- The choice of energy supply pathway for tumor cells, whether glycolysis or OXPHOS, is influenced by multiple factors.

## Open questions

- Is it crucial to identify the spectrum of tumor energy metabolism changes in clinical cancer management?
- How to establish specific clinical treatment plans through energy metabolism spectrum analysis?
- Tumor energy metabolism is flexible and diverse. Simply inhibiting one pathway is not significant. Is there a way to achieve a “kill two birds with one stone” treatment strategy for aerobic glycolysis and mitochondrial respiration in tumors under the guidance of energy metabolism spectrum screening?

## Introduction

Since Warburg’s discovery of aerobic glycolysis as a metabolic hallmark of cancer cells, extensive research has enhanced our comprehension of the metabolic alterations in cancer cells [1–3]. The Warburg hypothesis has been a dominant force in the field of cancer metabolism from the 1920s until the early 2000s. Recent studies have revealed a growing awareness of metabolic disparities between the culture conditions of extracorporeal tissues and the tumor microenvironment in vivo, suggesting that tumor cell metabolism extends beyond the Warburg effect [4], indicating intricate metabolic diversity within tumors and highlighting new avenues for exploration [5].

The reorganization of metabolism in tumor cells is vital for the initiation, proliferation, and advancement of cancer [6–8]. Thus, abnormal metabolism in tumors allows for new potential targets for cancer therapy, which highlights the importance of understanding tumor metabolism in order to develop effective treatment strategies. Catabolic pathways utilize carbon fuels—such as glucose, fatty acids, and glutamine—to generate adenosine triphosphate (ATP), which is utilized to sustain cellular functions and synthesize new cellular components [9]. In aerobic organisms, oxidative phosphorylation (OXPHOS) and glycolysis represent the primary pathways responsible for ATP generation. The utilization of these metabolic routes involves crucial tradeoffs, with glycolysis usually being more kinetically advantageous, while OXPHOS results in higher ATP production [10, 11]. The interplay between glycolysis and OXPHOS is crucial in determining the bioenergetics of tumors and is characterized by its complexity. Recent studies have demonstrated that energy supply pathways in tumor cells cannot be simply defined as glycolysis or OXPHOS. In addition to the metabolic heterogeneity and symbiosis observed in primary tumors, a metabolic shift between glycolysis and OXPHOS occurs during tumor progression [12–14]. Metabolic plasticity enables precise transitions between OXPHOS and glycolysis, enabling tumor cells to adapt to diverse microenvironments and finely regulate various biological activities [13, 15, 16]. What is intriguing is that the ratio of cell numbers between the two metabolic phenotypes has been established as a predictive factor for patient treatment response, physiological function, and survival [17].

This review centers on the relationship between glycolysis and OXPHOS, the two main pathways that supply energy to tumor cells, and the factors that influence the metabolic shift between them. From a static perspective, tumors harbor cells that rely on energy derived from both glycolysis and OXPHOS, often termed metabolic heterogeneity [18, 19], while metabolic symbiosis is defined by glycolysis and OXPHOS occurring simultaneously [20]. From a dynamic perspective, through tumor progression cancer cells undergo a metabolic shift between glycolysis and OXPHOS, known as metabolic plasticity. This process is influenced by various factors, such as the constantly changing tumor microenvironment, biological activities of tumor cells, and the effects of drug therapies [21–23]. Understanding the intricacies between glycolysis and OXPHOS are necessary to understand the mechanisms governing cancer control and may potentially unveil novel cancer treatments.

## Glycolysis or mitochondrial respiration

**Fig. 1:**
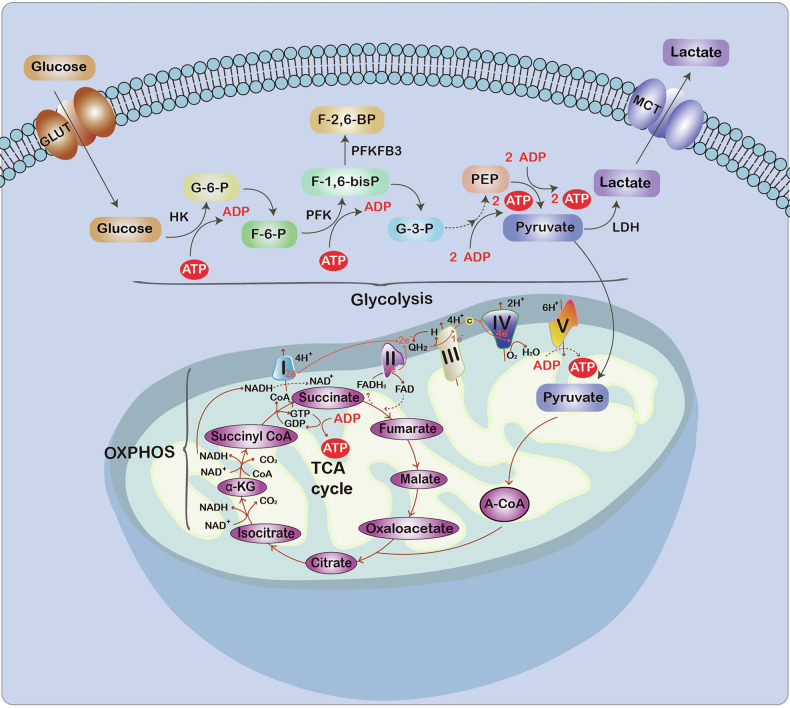
Schematic diagram of glycolysis and OXPHOS. Both glycolysis and OXPHOS serve as primary pathways for ATP production by directly supplying energy to cells.

OXPHOS and glycolysis are the primary pathways for ATP production, as illustrated in Fig. 1. In the 1920s, Warburg observed that tumor slices used glucose to generate lactate regardless of oxygen availability and surmised that “injury to respiration” necessitated a persistently elevated glycolytic flux to sustain proliferation, leading to the understanding that both mitochondrial dysfunction and aerobic glycolysis are defining characteristics of cancer [24, 25]. Subsequent research has validated the Warburg effect in both mouse models of cancer and in human cancer patients [5, 26]. In addition, ^18^Fluorodeoxyglucose positron-emission tomography in clinical settings has revealed that the glycolytic phenotype is a feature observed in most primary and metastatic human cancers [26].

By the mid-1970s, it was evident that OXPHOS dysfunction was not a common trait of all tumors [27]. Subsequent investigations revealed that the majority of cancer cells possess active mitochondria and still maintain the capacity to derive energy from OXPHOS and the tricarboxylic acid (TCA) cycle to support tumor growth [5, 15, 28, 29]. In fact, despite not respiring, most cancer cells harness the TCA cycle for energy and for synthesizing the metabolites required for their growth [5]. OXPHOS is also indispensable in delivering the energy and molecular components required for cancer cells in some tumor types [30].

Current evidence suggests that mitochondrial dysfunction and aerobic glycolysis are not always defining features of cancer cells, especially as tumors can vary metabolically and aerobic glycolysis does not necessarily indicate loss of OXPHOS [31]. Starting from a rudimentary understanding of cancer growth, the field of cancer metabolism has advanced to embrace the intricate metabolic complexities of tumors. In order to treat the different forms of cancer, it is imperative to have a thorough understanding of tumor metabolism.

## Static perspective

When we look at the energy metabolism of tumors from a static perspective, the energy supply within the tumor can be summarized as metabolic heterogeneity and metabolic symbiosis. Within a tumor, there are cells that rely on OXPHOS for energy, alongside cells that depend on glycolysis for energy, and cells that require both OXPHOS and glycolysis for energy—this is known as metabolic heterogeneity. Metabolic symbiosis refers to the interaction between cells that rely on both OXPHOS and glycolysis, through lactate transport and exchange, while simultaneously producing energy.

### Metabolic heterogeneity

**Fig. 2:**
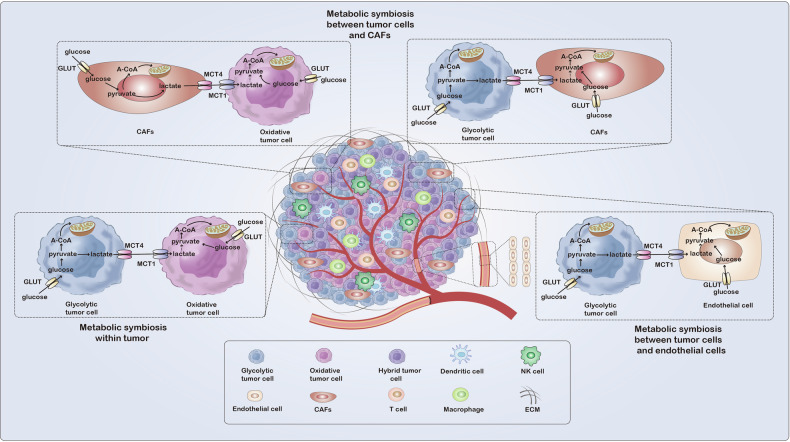
Metabolic heterogeneity of tumor. Within the tumor microenvironment, a variety of cell types coexist, including tumor cells, CAFs, endothelial cells, dendritic cells, T cells, macrophages, NK cells, and other cell types. Each cell type possesses unique metabolic requirements to fulfill specific functions. Moreover, tumor cells exposed to varied nutritional environments and perfusion levels exhibit diverse metabolic states, including glycolytic phenotype, oxidative phosphorylation phenotype, and hybrid state tumor cells. The connection between glycolytic phenotype cells and oxidative phenotype cells is referred to as “metabolic symbiosis”. The aforementioned circumstances contribute to metabolic heterogeneity within the tumor microenvironment.

Intratumoral heterogeneity arises from a number of causes [32]. Cancer cells reside within a microenvironment composed of various cell types, such as cancer-associated fibroblasts (CAFs), endothelial cells, immune cells like dendritic cells, T cells, macrophages, natural killer (NK) cells, and other cell types. Each cell type has unique metabolic demands that fulfill specific functions [19, 33]. Apart from the distinct metabolic requirements of each cell type, cancer cells are exposed to a unique nutrient environment and different levels of perfusion, receive distinct extracellular signals, and may originate from different cell types, thus potentially having a different metabolic state [6, 19]. Within the same tumor tissue, there may be a variety of cells, including those that rely solely on glycolysis, on OXPHOS, or in a hybrid state capable of utilizing both metabolic pathways [12, 34] (Fig. 2).

The metabolic heterogeneity within tumors is caused by the complex microenvironment present within the tumor. Blood vessels are formed in tumors to provide necessary nutrients and oxygen, yet vessel growth and blood supply become a limiting factor to support the increasing needs of a growing tumor. Thus, cancer cells are exposed to varying levels of blood-borne substances and perfusion-independent paracrine factors, creating a range of metabolic microenvironments that enable non-genetic tumor cell diversification. Glioblastoma (GBM) cells within this perivascular tier are characterized by intense anabolic metabolism and have a higher mitochondrial content and extensive use of OXPHOS [18]. Results from glucose tracing studies on non-small cell lung cancer (NSCLC) patients suggest that cancer cells in highly perfused areas utilize glucose to fuel glycolysis and OXPHOS, whereas cells in lowly perfused areas depend on other sources of carbon [35]. The metabolic characteristics of rare disseminated tumor cells in pleural effusion of lung adenocarcinoma patients involve both glycolysis and mitochondrial oxidation. Glycolytic cells display more mesenchymal-like expression and repressed expression of epithelial-related genes. In contrast, mitochondrial oxidation cells demonstrated elevated expression of epithelial-related genes and decreased expression of mesenchymal-related genes [17].

### Metabolic symbiosis

**Fig. 3:**
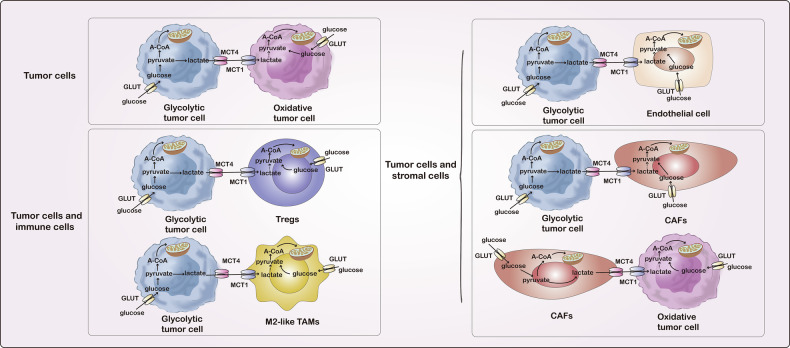
Metabolic symbiosis in tumors. The integration of OXPHOS and glycolysis occurs as a metabolic symbiosis between lactate-producing cells and lactate-consuming cells. The symbiotic relationship is present not just among hypoxic and normoxic cancer cells in the tumor, but also between cancer cells and cancer-associated stromal cells like endothelial cells and cancer-associated fibroblasts, as well as between cancer cells and immunosuppressive cells such as Treg and M2-like TAMs. Glycolytic cells release lactate via MCT4, whereas oxidative cells uptake extracellular lactate using MCT1 to replenish pyruvate and sustain the TCA.

The concurrent action of both OXPHOS and glycolysis for energy production, referred to as “metabolic symbiosis,” is a hallmark in cancer progressions [12]. Solid tumors exhibit metabolic symbiosis as a mix of lactate-producing cells and lactate-consuming cells, which occurs not only between hypoxic and normoxic cancer cells within the tumor, but also between cancer cells and cancer-associated stromal cells, as well as between cancer cells and immune cells [36–40] (Fig. 3). Two monocarboxylate transporters (MCT), MCT1 and MCT4, have been proposed to sustain this metabolic symbiosis through lactate transport and exchange. Glycolytic cells in tumors release lactate through MCT4, while oxidative cells in tumors absorb circulating lactate through MCT1 to regenerate pyruvate and fuel the TCA cycle [41].

Reports of a metabolic symbiosis between cells that generate and consume lactate have been observed in a variety of cancer types [42]. In vitro studies of pancreatic cancer cells revealed that hypoxic and normoxic cells can cooperatively support tumor growth, as the latter can up take lactate in the culture media to drive proliferation. Data from mouse pancreatic ductal adenocarcinoma (PDAC) experiments support these results, as expression of the MCT1 responsible for lactate uptake is increased in normoxic cancer cells and decreased in hypoxic cells. Patient-derived xenografts show a similar pattern of expression of MCT1, indicating that similar tumoral symbiosis may also be present in human PDAC [43]. In mouse breast cancer models, hypoxic tumor cells demonstrate high glucose uptake due to hypoxia-induced expression of glucose transporter type 1 (GLUT1) while hypoxia-induced expression of lactate exporter MCT4 enables efficient generation and export of lactate. Conversely, the lactate produced by the hypoxic tumor cells is taken up by the normoxic tumor cells and, when combined with oxygen from nearby blood vessels, is used to drive OXPHOS [44]. In addition, a study has indicated that metabolic symbiosis between tumor cells both close to and distant from blood vessels enables resistance to antiangiogenic treatments in both patient-derived renal cell carcinoma orthoxenograft models and clinical samples. The metabolic symbiosis phenotype was confirmed by indicative patterns of perivascular MCT1 and perinecrotic/hypoxic MCT4 areas that are mutually exclusive from each other. The mammalian target of rapamycin (mTOR) pathway regulates this metabolic patterning and its inhibition can successfully impede metabolic symbiosis in renal cell carcinoma orthoxenograft models [45].

Furthermore, metabolic symbiosis has been observed between tumor cells and stromal cells. Stimulated by hypoxia signals in deep tumors, metabolic symbiosis between tumor cells and endothelial cells (ECs) promotes the recruitment, proliferation, and migration of ECs, thus regulating the formation of abnormal tumor vascular networks [20]. Metabolic symbiosis has also been observed to be formed between cancer cells and CAFs and this symbiosis shifts based on oxygen levels and availability, extracellular metabolite concentration, and the presence of signalling molecules [46]. Upon contact between prostate cancer cells and CAFs, sirtuin 3-mediated regulation of hypoxia-inducible factor 1α (HIF-1α) and glycolytic phenotype is triggered, leading increased MCT4 and lactate secretion from CAFs. Simultaneously, prostate cancer cells upregulate MCT1 to facilitate lactate uptake, which is referred to as the ‘reverse Warburg effect' [47, 48]. In contrast, CAFs have also been demonstrated to use lactate exported from glycolytic cancer cells. Studies have demonstrated that cancer cells that are resistant to tyrosine kinase inhibitors have an enhanced glycolysis phenotype, as well as increased lactate production, whereas CAFs increased lactate uptake after MCT1 overexpression [49]. Through metabolic symbiosis, cancer cells and CAFs collaborate to promote cancer cell survival, growth, and resistance to treatment [50, 51].

Metabolic symbiosis exists between tumor cells and immune-suppressive cells as well. It has been shown in prior research that lactate can control the metabolic symbiosis between tumor cells and regulatory T cells (Tregs). Treg cells use cancer-cell-derived lactate not only to fuel the TCA cycle but also to produce phosphoenolpyruvate (PEP), which is essential for supporting Treg cell proliferation in the tumor. Treg cells can take up lactate through MCT1 and convert it into pyruvate for utilization in the mitochondrial TCA cycle. Deleting MCT1 in Treg cells showed that lactate uptake is not necessary for peripheral Treg cell function but is essential within tumors. This led to decreased tumor growth and improved response to immunotherapy [38]. Tumor-associated macrophages (TAMs) are a crucial component of the tumor microenvironment, playing a significant role in tumor development and drug resistance. The functions and polarization of macrophages are closely linked to metabolic changes. Generally, M1 macrophages typically use aerobic glycolysis, whereas M2 macrophages rely on oxidative metabolism, including increased OXPHOS and TCA [52]. Lactate derived from cancer cells promotes M2 polarization and also fuels the TCA cycle for M2-like TAMs [39, 40, 53].

## Dynamic perspective

The development of a tumor is a dynamic process, meaning that the energy supply pathways within the tumor cells are constantly evolving. In fact, the static state of tumor energy metabolism is just a temporary manifestation, while dynamic changes are the true reflection of tumor energy metabolism. Most cancer cells do not rely exclusively on either just glycolysis or OXPHOS and are instead able to dynamically shift their energy production in response to the tumor microenvironment, nutrient availability, and biogenic activities, often termed ‘metabolic plasticity’ [12, 54, 55]. Due to this plasticity, tumor cells are able to exploit two metabolic phenotypes to satisfy their energy requirements in the ever-changing tumor microenvironment, as well as during proliferation, migration, invasion, metastasis, and to acquire partial resistance to chemotherapy [15, 56, 57].

### Ever-changing tumor microenvironment

**Fig. 4:**
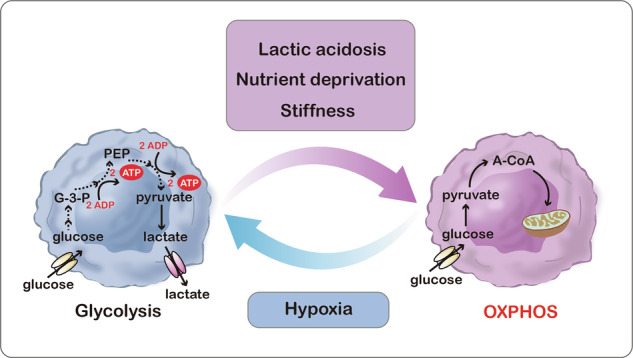
The impact of the ever-changing tumor microenvironment on energy metabolism. Lactic acidosis, nutrient deprivation, and stiff microenvironment promote tumor cell dependence on OXPHOS for energy supply, whereas under hypoxia tumor cells tend to favor glycolysis for energy production.

Throughout the various stages of carcinogenesis, cancer cells alter their metabolism to ensure survival in the tumor microenvironment (Fig. 4). Lactic acidosis, nutrient deprivation, hypoxia, and stiffness variation are frequent microenvironments in solid tumors [21, 58, 59].

Stringent growth conditions are thought to give rise to selection events that resemble bottlenecks, whereas less stringent conditions lead to phenotypic drift between cancer cells. Recent studies have demonstrated that environments characterized by combinations of hypoxia, acidosis, low glucose, and starvation create a potent selective pressure for breast cancer cells to shift to aerobic glycolysis. This shift towards a Warburg phenotype is linked to transcription factor Krüppel-like factor 4 (KLF4) expression. It was found that KLF4 regulates all genes responsible for lactate production rate. Examination of KLF4 distribution in ductal carcinomas in situ of the breast specimens showed that many areas displayed elevated levels of KLF4 at the center of the duct, where access to nutrients is restricted. This results in a strong selection pressure characterized by increased acidosis and decreased oxygen levels [60]. In contrast, GBMs have elevated rates of glycolysis, lactic acidosis, and glucose deprivation. However, studies in U251 GBM cells have demonstrated that lactic acid can decrease glycolysis and increase OXPHOS by diminishing the expression of HIF-1α and augmenting c-myelocytomatosis oncogene (c-MYC) and nuclear respiratory factor 1 (NRF1) expression [21]. Studies of five different cancer cell lines (HeLa, AGS, RKO, A549, and SK-HEP-1) revealed that lactic acidosis significantly decreases glycolysis by 79-86%, while increasing OXPHOS by 177–218%, establishing OXPHOS as the primary ATP production pathway in these cells. Consequently, cancer cells exhibiting the Warburg effect phenotype are susceptible to OXPHOS inhibitors under lactic acidosis, thereby broadening the therapeutic potential of such inhibitors [61].

When glucose is deprived and replaced with galactose, a metabolic shift occurs in GBM cells from glycolysis to OXPHOS, leading to mitochondrial fission. This process relies on NF-κB-inducing kinase (NIK) and dynamin-related protein 1 (DRP1) [62]. Under serum-depleted conditions, U251 GBM cells were observed to reduce glycolysis and increase OXPHOS in order to resist cell death via the HIF-1α/C-MYC pathway [63]. The metabolic characteristics of melanoma cells change during tumorigenesis, utilizing a combined glycolysis/OXPHOS metabolic phenotype that gives tumor cells a distinct advantage. Similar to GBM cells, when deprived of glucose, melanoma cells modify their metabolism to rely more heavily on OXPHOS and less on glycolysis [64]. In addition, ovarian cancer cell clones that were resistant to glucose deprivation were able to survive and overcome the stress caused by glucose starvation by increasing their OXPHOS and uptake of both pyruvate and lipids. In contrast, those that were sensitive to glucose deprivation failed to adapt and consequently died [65].

Hypoxic conditions force tumor cells to alter their metabolism, transitioning from OXPHOS to anaerobic glycolysis [66]. Under hypoxic conditions, HIF-1α serves as the primary sensor of oxygen deprivation, inducing the overexpression of pyruvate dehydrogenase kinase 1 and suppressing pyruvate dehydrogenase, thereby inducing anaerobic glycolysis [16]. Talasila et al. revealed that patient biopsies had higher expression of lactate dehydrogenase A and glucose transporter 1 in hypoxic areas compared to the invasive edge and lower-grade tumors. Subsequent mitochondrial respiratory chain analysis indicated decrease complex I activity in angiogenic xenografts and hypoxic regions of GBM samples when compared to the levels in invasive xenografts, non-hypoxic GBM regions, and lower-grade tumors [67].

Moreover, the stiffness in heterogeneous microenvironment influences metabolic heterogeneity in breast cancer cells. Correlative stiffness maps of distinct locations within breast tumor biopsies reveal that stiffness increases from the center to the periphery. Cancer cells shift their metabolic pathways from glycolysis to OXPHOS and fatty acid metabolism in response to stiff microenvironments, changing the expression of genes connected to mitochondrial respiration and increasing pathways associated with OXPHOS and fatty acid metabolism [59].

### Biogenic activities

#### Proliferation

Tumor cell subpopulations that are quiescent rely less on glycolysis and more on OXPHOS, as well as exhibiting increased expression of mitochondrial respiratory components. These subpopulations contain stem-like subclones that are generated following oncogene ablation in vivo, in addition to circulating tumor cells (CTCs) [64, 68]. Cancer stem cells from various types of cancer, such as those found in the pancreas and cholangiocarcinoma, exhibit a strong reliance on OXPHOS for their energy production [69, 70].

**Fig. 5:**
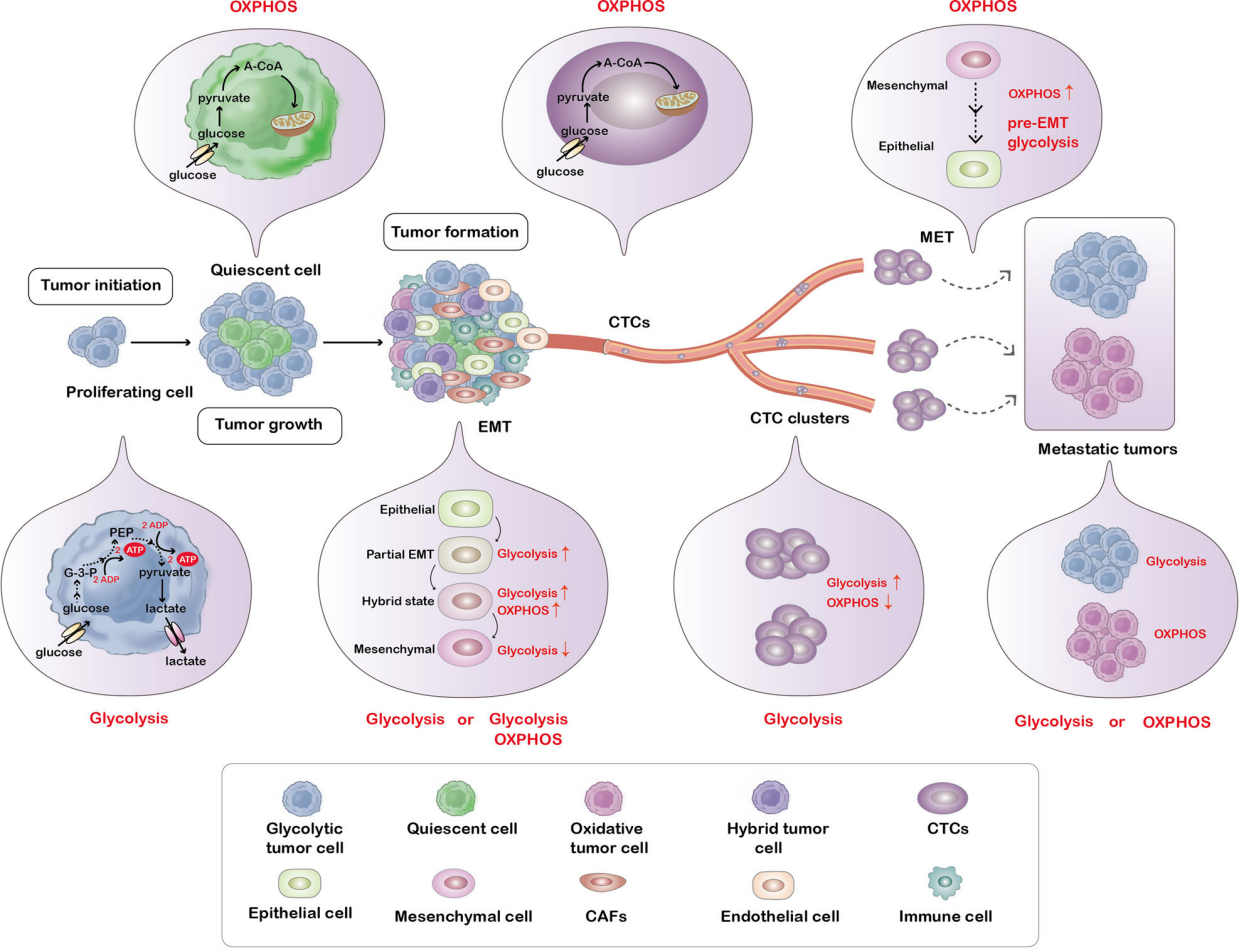
Alterations in energy metabolism during the biological processes of tumor cells. Proliferating cells exhibit a preference glycolysis rather than OXPHOS, whereas quiescent cells demonstrate reduced reliance on glycolysis and increased dependence on OXPHOS. The metabolic flexibility during tumor metastasis confers a survival advantage, as cancer cells undergo numerous dynamic changes and metabolic adaptations. The process of EMT is closely associated with upregulated glycolysis or heightened levels of both glycolysis and OXPHOS activity. While traveling to distant organs, CTCs utilize OXPHOS as their primary energy source. The formation of clusters among CTCs is correlated with their ability to colonize distant metastatic sites, as these clusters exhibit enhanced glycolysis and decreased dependence on OXPHOS. The induction of MET significantly increases OXPHOS levels beyond those observed pre-EMT, solely restoring the glycolytic rate in cancer cells to its pre-EMT state. The metabolic dependencies of tumor cells at metastatic sites rely on the synchronized metabolic pathways between cancer cells and the secondary site microenvironment. Some metastatic tumor cells rely on OXPHOS, while others depend on glycolysis.

In contrast, proliferating cells have distinct carbon and energy metabolisms compared to quiescent cells [15]. Proliferating tumor cells favor a transition towards glycolysis over OXPHOS when exposed to oxygen, whereas slow-cycling tumor cells may prioritize mitochondrial respiration as their main energy source [4] (Fig. 5). Proliferating cells only slightly increase their ATP consumption in comparison to the requirement of precursor molecules and reducing equivalents, such as nicotinamide adenine dinucleotide phosphate (NADPH), that are derived from glucose metabolism. However, glucose metabolism is significantly limited by the TCA cycle, which produces nicotinamide adenine dinucleotide (NADH) and ATP. Thus, excess pyruvate is converted to lactate by proliferating cells in order to avoid NADH accumulation in the cytoplasm and to prevent ATP generation from hindering glucose metabolism in the cytoplasm [15]. Further, glycolytic intermediates produced during glycolysis are utilized in various biosynthetic reactions during cell proliferation. For instance, the glucose-6-phosphate intermediate of glycolysis may be diverted to the pentose phosphate pathway to produce ribose-5-phosphate and NADPH, which are necessary for nucleotide synthesis. Meanwhile, dihydroxyacetone phosphate is converted to glycerol-3-phosphate, which is used for the biosynthesis of phospholipids, an essential component of cellular membranes [15, 22]. Accordingly, the rate-limiting enzymes in the branching pathways of glycolysis are often increased in proliferating cancer cells [15]. These data indicate that glycolysis is advantageous when cells are proliferating.

Pyruvate kinase M (PKM), a glycolytic enzyme, can be spliced in two different ways to create either the PKM1 or PKM2. PKM2-expressing cells convert more pyruvate to lactate, whereas PKM1 cells metabolize more pyruvate in the mitochondria [71, 72]. David et al. established that the PKM splicing regulatory proteins might be regulated by a regulatory system related to proliferation [73], and PKM2 is selectively expressed in proliferating cancer cells and may create a metabolic environment that is favorable for cell proliferation [72]. Thus, the growth of tumor cells was significantly decreased when PKM2 was replaced with PKM1 [73]. In addition, decreased mitochondrial activity, indicated by high expression of uncoupling protein 2, drives cancer cell proliferation with a concomitant metabolic shift from mitochondrial OXPHOS to glycolysis [22, 74].

#### Migration and metastasis

The energy supply for migration and metastasis is incredibly intricate. Some cancer researchers have proposed that cells must make a choice between “grow or go”, as proliferation and migration are incompatible due to their shared resource consumption [75]. As such, metabolic signaling can be distinct when it comes to migration and proliferation, where migratory/invasive cancer cells have a preference for mitochondrial respiration and increased ATP production [64]. Facilitating mitochondrial respiration in kidney cancer cells has been shown to boost metastasis, whereas suppressing complex I of the electron transport chain can decrease metastasis [76]. By enhancing mitochondrial respiration, phospholipase C gamma 2, found in extrachromosomal circular DNAs, aids in the metastasis of non-small cell lung cancer [77]. However, other studies have indicated that the production of ATP through glycolysis, instead of mitochondrial OXPHOS, supports cell migration [78]. Studies have indicated that an increase in glycolysis in human osteosarcoma 143B cells with mitochondrial dysfunction leads to increased cell migration, and that malate dehydrogenase 1 may be involved in sustaining glycolytic ATP production [79]. Elevated levels of plasminogen activator inhibitor 1 in migratory cancer cells have been linked to the promotion of glycolysis in triple-negative breast cancer cells [80]. Consequently, it has been suggested that cells are able to rapidly respond to energy requirements through glycolysis and can precisely adjust the energy requirement within the cell through mitochondria rearrangement [56].

In fact, the process of cancer cell metastasis is driven both by OXPHOS and glycolysis, rather than relying solely on one pathway. This metabolic flexibility provides an advantage during metastasis as cancer cells undergo numerous dynamic changes and metabolic adaptations (Fig. 5). The process of cancer metastasis entails the following sequential events: (I) Cancer cells undergo epithelial-to-mesenchymal transition (EMT) to escape from the primary tumor and enter the bloodstream; (II) Post-EMT, these cells extravasate into the blood, assuming the identity of CTCs, and forming CTC clusters; (III) Upon successfully anchoring at their destination, cancer cells commonly undergo mesenchymal-to-epithelial transition (MET); (IV) Ultimately, metastatic tumors form [16].

The process of EMT is closely associated with upregulated glycolysis, where enzymes like hexokinase 2, phosphofructokinase (PFK), and PKM2 (key participants of glycolysis), enhance glycolytic flux and promote EMT [81]. Downregulation of hexokinase 2 and PFK results in decreased glycolysis and suppressed EMT [82]. Inhibition of PKM2 leads to a reshaping of the tumor immune microenvironment and a reversal of EMT in colorectal cancer and liver metastases by reducing lactate levels and suppressing transforming growth factor-β signaling. Transforming growth factor-β is known to stimulate EMT and contribute to the formation of nuclear dimer PKM2, which in turn enhances E-cadherin expression [83]. The reprogramming of glucose metabolism through glycogen phosphorylase L-mediated mobilization of glycogen promotes the EMT phenotype, thereby enhancing metastasis in PDAC by fueling glycolysis [84]. However, the luminal clusters in primary breast cancer undergo a shift towards mesenchymal gene expression, resulting in the development of epithelial/mesenchymal subpopulations that are sustained by both OXPHOS and glycolysis [85]. Evidence from in vitro and in vivo studies indicate that tumor cells undergoing partial EMT exhibit elevated glycolysis levels, transitioning to a hybrid state with concurrent epithelial and mesenchymal characteristics, alongside heightened glycolysis and OXPHOS activity. In this hybrid state, a complete mesenchymal phenotype may emerge with lowered glycolytic levels. Conversely, a decrease in both glycolysis and OXPHOS could induce a transition to a quiescent mesenchymal-like state [57].

CTCs have the ability to seed distant metastases by disseminating via the bloodstream. During their journey to remote organs, CTCs rely on OXPHOS for energy, a mechanism that is facilitated by increased expression of peroxisome proliferator-activated receptor gamma coactivator 1α (PGC-1α). CTCs originating from melanoma, prostate cancer, lung adenocarcinoma, and breast cancer display a notable increase in the enrichment of OXPHOS pathway genes compared to their respective tumor counterparts [86, 87]. The ability of CTCs to colonize distant metastatic sites is linked to the formation of clusters. Within these clusters, cells exhibit increased glycolysis and reductive glutamine metabolism, while also reducing their dependence on OXPHOS to lower reactive oxygen species (ROS) production and maintain fitness. Despite this metabolic shift, they still maintain their capability for OXPHOS [88].

Upon successful anchoring at their metastatic site, cancer cells frequently experience a MET, adjusting their metabolic profile to align with the local microenvironment for enhanced survival and increased proliferation [89, 90]. The transcriptional profile of MET is not a simple mirror image of EMT, as tumor cells maintain a transcriptional “memory” after undergoing reversible EMT. The induction of MET significantly increased the basal and maximal oxygen consumption rates above the pre-EMT levels, while only restoring the pre-EMT glycolytic rate in LNCaP cells [89].

The successful spread of cancer cells to distant sites, known as metastasis, relies on the synchronization of metabolic pathways between the cancer cells (referred to as the “seed”) and the microenvironment of the secondary site (referred to as the “soil”). Cells with metabolic flexibility are thus more adept at infiltrating multiple locations with higher efficiency [16]. For example, multisite metastatic breast cancer 4T1 cells exhibit elevated glycolytic and TCA cycle rates compared to non-metastatic cell lines 67NR. They also demonstrate enhanced flexibility in switching between glycolysis and OXPHOS in response to environmental stimuli. Tumors originating from 4T1 cells exhibit the ability to modulate both glycolysis and mitochondrial metabolism according to the specific microenvironment of individual metastatic sites [91, 92]. Liver-metastatic breast cancer cells increase glycolysis through HIF-1α/pyruvate dehydrogenase kinase-1, while bone- and lung-metastatic cells have higher levels of OXPHOS express PGC1α at a higher rate and have increased oxidative stress [91–93]. In melanoma, brain metastases demonstrate a higher level of OXPHOS than both lung metastases and primary tumors, and the suppression of OXPHOS has been observed to decrease the formation of brain metastases [94]. It is uncertain whether a transformation in metabolic phenotype in the primary tumor is a factor in choosing the metastatic site, or if metabolic reprogramming transpires at the secondary site. Further studies could reveal how metabolic plasticity and flexibility of cells impact migration through microenvironments and the various stages of metastasis. Gaining insight into how metabolic flexibility changes during metastasis is essential for devising effective treatments.

Furthermore, there are certain unique circumstances present during the process of tumor cell migration. Depending on the cell type and the surrounding environment, cells can migrate either by themselves or as collective groups. Collective migration is characterized by a changing invasive front, with leader cells requiring more metabolic energy than follower cells to successfully penetrate the extracellular matrix (ECM) due to their increased contractility and strain energy [95]. Studies have revealed that, during the collective migration of breast cancer cells, the leader cells at the front of the invasion display a higher glycolytic flux than follower cells, while the leader cells in lung cancer have been observed to possess a higher OXPHOS than follower cells [95, 96], suggesting a tumor type-specific bioenergetic switch of metastatic cancer cells. However, the regulation of this balance between glycolysis and OXPHOS in leader cell behavior and the role of the mechanical microenvironment in this process remain unknown. Cancer cell migration is also associated with cellular energy status, as when oxygen and glucose supply remain uninterrupted, invading cancer cells adapt their energy homeostasis by utilizing a combination of OXPHOS and glycolysis to sustain their migratory capabilities in reaction to microenvironment mechanical and chemical stimuli. OXPHOS delivers energy specifically to the high-energy areas of the cell, while mitochondria move towards the cell’s forefront to aid in cytoskeletal functions, membrane extension, and focal adhesion formation. Glycolysis that occurs in close proximity to cytoskeletal operations fuels migration by generating ATP. In the presence of energy deprivation and hypoxia, ATP production mainly relies on glycolysis [57].

### Pharmacologic perturbations

The metabolic plasticity of tumor cells is a major factor in cellular drug resistance. Melanoma cells are characterized by a remarkable metabolic plasticity, which allows them to both adjust to hostile tumor microenvironments and render them partially resistant to chemotherapy, thus providing them with a survival benefits in treatment-related situations [97]. The activity of the serine/threonine-protein kinase B-Raf/mitogen activated protein kinase (BRAF/MAPK) pathway has been observed to regulate the switch from OXPHOS to glycolysis in melanoma cells and can be targeted by inhibitors of BRAF or MEK (MAPK and extracellular-regulated kinase (ERK) kinase; an upstream activator of MAPK). Patients treated with inhibitors of BRAF or MEK exhibited a metabolic transition from glycolysis to OXPHOS, alongside an adaptive resistance to these inhibitors, increased mitochondrial content, activity, and oxidative capacity [12, 97]. Five ovarian cancer cell lines, C200, PEO4, OVCAR5, OVCAR3, and OVSAHO, which are resistant to cisplatin, have been observed to demonstrate metabolic flexibility between glycolysis and OXPHOS [23, 98]. Hence, a better understanding of the mechanisms of and reducing metabolic plasticity in cancer cells is necessary for future cancer chemotherapy research.

## Other factors affecting tumor metabolic pathways

### Genotype and tissue of origin

Various oncogenic drivers in the same tissue of origin can cause different metabolic changes, while the same oncogenic driver in different tissues of origin can lead to varied metabolic alterations. Thus, metabolic profiles of tumors are contingent upon both the genotype and tissue of origin [5].

One source of differentiation is different levels of gene expression in cells derived from the same source tissue, which leads to different metabolic conditions. Sancho et al. revealed that pancreatic cancer cells with higher MYC expression and lower PGC-1α expression rely more heavily on glycolysis, whereas those with increased PGC-1α expression and reduced MYC expression demonstrate a greater stem-like characteristic and are more reliant on OXPHOS. Cells with an intermediate phenotype expressed PGC-1α and MYC at intermediate levels and produced ATP via both glycolysis and mitochondrial OXPHOS. These cells were subsequently resistant to metformin treatment and were able to adapt to OXPHOS inhibition by activating glycolysis [16]. Yu et al. demonstrated that HIF-1α and AMP-activated protein kinase (AMPK) activities were inversely related in many cancer cells, where cells with high HIF-1α and low AMPK expression relied more on glycolysis and those with low HIF-1α and high AMPK expression relying on OXPHOS. Additionally, other cancer cells have been observed to use HIF-1α and AMPK as core genetic circuitry for metabolic plasticity, which enables them to simultaneously increase glycolytic and OXPHOS capacities [99, 100]. Separately, colorectal adenoma cells that are reliant on OXPHOS display elevated levels of DNA methyltransferase 1 and reduced levels of N-methyltransferase expression. By overexpressing N-methyltransferase and silencing DNA methyltransferase 1, the OXPHOS-inhibited cell lines shifted from OXPHOS to glycolysis [101]. The primary role of crucial metabolic regulators in cancer may be to enhance metabolic adaptability, allowing cancer cells to respond to changing conditions as the disease advances.

The same oncogenic driver, when present in different tissues of origin, can result in varied metabolic changes. MYC expression is elevated in colon cancer regardless of the tumor stage, and this has been linked to the expression of 231 different metabolic genes (i.e., glycolysis, the pentose phosphate pathway, purine and pyrimidine biosynthesis, fatty acid biosynthesis, one-carbon metabolism, and several metabolite transporters). Simultaneously, these cells have decrease expression in genes related to the TCA cycle and fatty acid oxidation. In contrast, prostate tumors with high MYC levels do not display increase expression in glycolytic genes, while there was increased fatty acid biosynthesis pathway gene expression when compared to normal prostate tissues [102].

Additionally, there are other gene expressions and modifications associated with energy metabolism. The tumor suppressor gene p53 inhibits glycolysis and enhances mitochondrial OXPHOS through a cascade of downstream targets to counteract the Warburg effect [103]. Genes whose absence leads to an increase in OXPHOS activity are enriched in components of the pre-mRNA splicing machinery, specifically in U1 snRNP subunits. One of these genes is LUC7L2, which codes for a U1 small nuclear ribonucleoprotein (U1 snRNP) subunit essential for pre-mRNA splicing and gene expression. The depletion of LUC7L2 and U1 snRNP subunits leads to a transition from glycolysis to OXPHOS in chronic myeloid leukemia cells [10]. Moreover, RNA modification is involved in the metabolic reprogramming that occurs during cancer metastasis. In human oral cancer, RNA modifications, specifically the absence of 5-methylcytosine, lead to heightened glycolysis and impaired mitochondrial function in cancer cells. Notably, these alterations do not impact cell viability or the growth of primary tumors [55].

### Metabolites and enzymes

Metabolites associated with the TCA and enzymes related to both TCA and glycolysis pathways also impact tumor energy metabolism. Citrate is a crucial metabolite in the TCA cycle, serving as a vital intermediary in metabolic pathways [104]. Acting as an activator for gluconeogenesis and an inhibitor for glycolysis, the maintenance of low citrate levels is crucial for highly proliferating cancer cells. The main regulators of glycolysis, PFK1 and PFK2, are directly inhibited by citrate. Cancer cells that predominantly utilize glycolysis benefit from reduced citrate levels, supporting the Warburg effect. Conversely, cells reliant on OXPHOS generate more citrate through fatty acid oxidation, which is promptly converted into acetyl-CoA and oxaloacetate [105]. Succinate, an intermediate in the TCA cycle, is normally contained within the mitochondrial matrix [106]. The mutation of succinate dehydrogenase (SDH) subunits (SDHD and SDHB) in hereditary tumors like paraganglioma, or the reduction of SDHB expression in cancer, causes succinate to accumulate in the matrix. Excessive succinate can leak into the cytoplasm and is subsequently secreted into the extracellular space. Cytosolic succinate stabilizes HIF-1α by inhibiting prolyl hydroxylase domain, which is essential for HIF-1α degradation through the ubiquitin-proteasome system. HIF-1α is responsible for transcribing genes that play crucial roles in glycolysis, angiogenesis, and cancer metastasis [107]. Oxaloacetate also serves as an intermediary in the TCA cycle and oxaloacetate treatment in human hepatocellular liver carcinoma cells inhibits glycolysis and enhances OXPHOS, ultimately inducing apoptosis in cancer cells [108].

Apart from metabolites, enzymes related to metabolism also play a role in influencing tumor metabolic pathways. Isocitrate dehydrogenase (IDH) is a pivotal enzyme within the TCA cycle that is responsible for the reversible conversion of isocitrate to α-ketoglutarate (α-KG) [109]. IDH1/2 mutations occur in low-grade glioma, acute myelogenous leukemia, and secondary GBM [110]. Gliomas with IDH1 mutations exhibit reduced glycolytic flow and heightened TCA cycle function [111, 112]. In sensitive leukemia cells, mutant IDH1 enzymes facilitate the conversion of α-KG to R-2-hydroxyglutarate, inducing glycolytic inhibition and reducing lactate and ATP levels, while displaying minimal impact on mitochondrial respiration in both sensitive and resistant cells [113]. SDH, also referred to as mitochondrial complex II, is an enzyme located in the mitochondria that plays a crucial role in both the TCA cycle and the electron transport chain [114]. SDH impairment can lead to compromised mitochondrial activity, affecting ATP synthesis and energy balance. This deficiency can result in PKM2 overexpression and increased glycolytic activity in renal cell carcinoma [115]. Fumarate hydratase (FH) is also a crucial enzyme required for the TCA cycle, as cells deficient in FH respond to mitochondrial dysfunction through a cascade of compensatory metabolic adjustments, including upregulation of glycolysis, enhanced transcription of glycolysis-related genes, and inhibition of pyruvate dehydrogenase [116]. PKM2, the rate-limiting enzyme in glycolysis, plays a crucial role in regulating metabolic processes. Silencing PKM2 leads to a concurrent decrease in glycolytic activity and OXPHOS in both H1299 (human lung cancer-derived) and HepG2 (human liver cancer-derived) cells. This underscores the interconnectedness of glycolysis and OXPHOS, emphasizing the pivotal function of PKM2 in cancer cell metabolism. PKM2 interacts with MFN2 to enhance mitochondrial fusion and OXPHOS while reducing glycolysis. The binding between PKM2 and MFN2 is strengthened through mTOR-induced phosphorylation of MFN2 [117].

### Mitochondrial function

Mitochondrial function serves an important role in shaping the metabolism spectrum of tumors, especially between malignant cells from the same tumor and non-malignant cells of the same type, which is attributed to variations in OXPHOS gene expression. OXPHOS activity and regulation variability has been identified as the primary cause of metabolic and functional heterogeneity, and correlates with glycolysis and hypoxia response. This fluctuation in OXPHOS activity suggests that this pathway could be utilized for adapting to environmental conditions [19, 118]. Abnormalities in OXPHOS and mitochondrial biogenesis can lead to a transition in energy production from oxidative to aerobic glycolysis, possibly reshaping the metabolic landscape of tumors [119]. In NSCLC tumor subtype, various functional OXPHOS signals are identified. NSCLC tumors that have heightened OXPHOS and fatty acid oxidation exhibit peridroplet mitochondrial networks, which are distinguished by mitochondria surrounding lipid droplets. This phenomenon is accompanied by notable enhancements in the respiratory activities of complex I and complex II, increased crista density, and an elevated basal oxygen consumption rate. In contrast, the NSCLC tumor subtype exhibiting low OXPHOS rates demonstrates a regulation of high glucose flux, which influences the perinuclear positioning of mitochondria, structural alterations in cristae, and the respiratory capacity of mitochondria [120]. Moreover, other reports have indicated a correlation between mitochondrial fragmentation and metabolic reprogramming, causing an increase in glycolysis and a decrease in OXPHOS [121].

## Discussion

The evolving landscape of tumor metabolism research is reshaping our understanding of its complexity and the molecular mechanisms at play, prompting a redefinition of fundamental principles in this field. Enhancing our comprehension of the metabolic factors that facilitate tumor growth within the microenvironment is essential for developing and effectively implementing targeted metabolic inhibitors and diagnostic imaging techniques in clinical oncology [122]. The study of glycolysis and OXPHOS, both essential pathways that directly fuel cancer cells, holds particular significance [10]. As research on cancer cell metabolism progresses beyond the Warburg effect, it is no longer sufficient to merely characterize cancer cells by mitochondrial dysfunction and aerobic glycolysis [30]. Altered energy metabolism, which should be a recognized hallmark of cancer, serves as a distinctive biochemical signature of cancer cells. It is noteworthy that metabolite expression patterns vary among different tumors, influenced by a number of factors, including tumor origin, oxygen levels, energy supply, and blood flow to tumor cells [18, 102, 120]. Consequently, a detailed analysis of the energy metabolism traits is crucial when targeting energy production pathways in specific tumors, which can be attained using effective metabolic detection technologies.

This review delves into the relationship between glycolysis and OXPHOS from both static and dynamic perspectives. Tumors display metabolic heterogeneity and metabolic symbiosis in static terms. Heterogeneity in energy metabolic phenotypes is observed among constituent cells within an individual tumor [123]. The metabolic symbiosis that exists between tumor cells and other cells, such as CAFs, or tumor cells between glycolysis and the oxidative regions, is regarded as a fundamental determinant of cancer malignancy [124, 125]. From a dynamic perspective, cancer cells either transition between glycolytic and OXPHOS-derived energy states or exhibit both glycolysis and OXPHOS concurrently (i.e., metabolic plasticity). Various factors influence the transition between glycolysis and OXPHOS, including the tumor microenvironment (e.g., lactic acidosis, glucose deprivation, hypoxia, and stiffness), biological processes, and drug treatments. The metabolic plasticity of tumor cells plays a crucial role in the progression of tumors, such as proliferation, invasion, and metastasis. During tumor cell proliferation, even in the presence of oxygen, tumor cells preferentially choose glycolysis as their main source of energy [4, 15]. During the invasion of tumors, leading cells at the invasion front require a higher metabolic capacity than following cells to successfully penetrate the ECM. In different types of tumors, leading cells at the invasion front exhibit varying metabolic profiles. For instance, in breast cancer, leading cells at the invasion front demonstrate higher glycolytic flux compared to following cells, whereas in lung cancer, leading cells show higher OXPHOS than following cells [95, 96]. As depicted in Fig. 5, the progression of tumor metastasis does not solely depend on a single pathway; instead, it is driven by both OXPHOS and glycolysis [16]. This metabolic flexibility provides cancer cells with an advantage during the process of metastasis, as they undergo significant dynamic changes and metabolic adaptations. In conclusion, at different stages of tumor development, the energy phenotype of tumor cells varies, necessitating the targeting of different energy metabolism pathways during treatment. Therefore, when treating tumors at different stages, it is advisable to utilize metabolic biomarkers to identify metabolic-dependent subgroups. Cell subpopulations with different metabolic phenotypes require precise combination therapy, such as the rational pairing of glycolysis inhibitors with OXPHOS inhibitors. Clinical practice in the future will focus on personalized treatment with dynamic adjustments based on real-time metabolic profiles of individual tumors to effectively halt tumor progression.

In conclusion, the relationship between glycolysis and OXPHOS in tumor development conforms to the category of dialectical materialism: relatively static, absolutely dynamic. The steady state of tumor energy metabolism is temporary, while the dynamic changes in energy metabolism are eternal. In our previous research, we proposed the importance of screening for energy metabolism in cancer treatment [126]. In this review, we put forward a more in-depth perspective: a one-time screening is insufficient to characterize tumor energy phenotype, necessitating dynamic monitoring and management of tumor energy metabolism. At various stages of tumor progression, there are alterations in energy metabolism that necessitate diverse therapeutic approaches. Employing dialectical materialism to guide medical practice necessitates a high level of attention to tumor energy metabolism phenotype screening during the treatment process, as well as the ability to adapt treatment plans based on changes in energy metabolism phenotype in order to decrease the tumor’s resistance to treatment strategies.
